# Optimization Criteria and Efficiency of a Thermoelectric Generator

**DOI:** 10.3390/e24121812

**Published:** 2022-12-13

**Authors:** V. H. Juárez-Huerta, N. Sánchez-Salas, J. C. Chimal-Eguía

**Affiliations:** 1 Departamento de Física, Escuela Superior de Física y Matemáticas, Instituto Politécnico Nacional, UP Zacatenco, Ciudad de México CP 07738, Mexico; 2 Laboratorio de Simulación y Modelado, Centro de Investigación en Computación, Instituto Politécnico Nacional, Av. Juan de Dios Batiz s/n UP Zacatenco, Ciudad de México CP 07738, Mexico

**Keywords:** endoreversible limit, exoreversible thermal engine, finite time thermodynamics, thermoelectric generator

## Abstract

The efficiency of a thermoelectric generator model under maximum conditions is presented for two optimization criteria proposed under the context of finite-time thermodynamics, namely, the efficient power criterion and the Omega function, where this last function represents a trade-off between useful and lost energy. The results are compared with the performance of the device at maximum power output. A macroscopic thermoelectric generator (TEG) model with three possible sources of irreversibilities is considered: (i) the electric resistance *R* for the Joule heating, (ii) the thermal conductances $K_{h}$ and $K_{c}$ of the heat exchangers between the thermal baths and the TEG, and (iii) the internal thermal conductance *K* for heat leakage. In particular, two configurations of the macroscopic TEG are studied: the so-called exoreversible case and the endoreversible limit. It shows that for both TEG configurations, the efficiency at maximum Omega function is always greater than that obtained in conditions of maximum efficient power, and this in turn is greater than that of the maximum power regime.

## 1. Introduction

The pioneering works of T.J. Seebeck [1,2,3] at the beginning of the 19th century gave rise to the study of thermodynamics in thermoelectric phenomena. The fundamental Seebeck’s idea published in 1821 of generating electricity by applying heat into the junction of two distinct materials marked a new era in the study of several thermoelectric phenomena; see, for instance, Peltier [4], Thomson [5], etc. These authors placed the theory foundations of the thermoelectricity as a non-equilibrium phenomenon, but the physical and mathematical tools of that time were insufficient to further describe it.

On the other hand, at the end of the 20th century with the pioneering work of Curzon and Alhborn, the newborn finite-time thermodynamics (FTT) obtained one of the successful models to describe heat engines under more realistic conditions [6,7,8,9,10], which is something that the classical equilibrium thermodynamics (CET) had not yet achieved. In this sense, the CET had only shown what the upper limits were for some variables related to the process itself, such as the efficiency.

As a result of having incorporated more realistic operating conditions into the study of thermal engines, it was possible to obtain models whose results are more in line with reality [11,12,13,14]. Many of these models that have emerged from the FTT are being developed in the context of finding optimization criteria that provide insights on how to combine state variables that could influence the efficiency of the thermal engines. Among the most important can be found: (a) minimization of entropy generation [15,16,17,18], (b) maximization of the power output [19], (c) maximization of the ecological function [20], (d) maximization of the Omega function [21], (e) maximization of the saving function [22], (f) maximization of the efficient power [23], etc. The loop-shaped-type curves must be emphasized as one of the intuitive properties that have been incorporated into the FTT models, in which analysis of the power versus efficiency shows how irreversibilities play an important role in the functioning of the engine.

The success of the FTT was outstanding in areas as diverse as quantum physics [24,25], biology [26,27,28,29], chemistry [30,31], engineering [32,33], etc. In all of these, the common procedure was to model the different systems by means of the FTT, trying to obtain the optimization criteria that explain the efficiency shown in the real systems.

It is in this context that the thermoelectric generators (TEGs) began to be studied as thermal engines that directly convert heat into electricity via the Seebeck effect, and have the advantage of allowing each of their sources of irreversibility to be modeled separately, and in a simple way. It is important to note that TEGs have a very low efficiency in comparison to traditional heat engines, and looking for mechanisms to improve it could be a key aspect for many technological applications. One example in this direction is some recent energy harvesting technologies, such as triboelectric generators (TENGs) [34], piezoelectric generators [35], and thermoelectric generators (TEGs) [36,37], which have been developed to convert human biomechanical energy into electricity. The energy needed to run these devices is around 1% of the 100 W that a human generates daily due to mechanical activities [38].

The human body as a thermal source would be sufficient to generate this amount of energy; however, achieving this efficiency could be an engineering challenge because the area that could be covered by these TEGs is finite [38], and then it becomes important for these devices to maximize the efficiency. It has been shown that one effective way to improve this efficiency is to develop thermoelectric materials with a high figure of merit zT [18,38]. For instance, the highest zT that has been achieved for inorganic materials is 2.2, while for organic materials the highest zT is 0.75 [38]. The value of zT is mainly due to low thermal conductivity and high electrical conductivity that assist in the transfer of electrons during the thermoelectric process [39]. In some cases, it is not the figure of merit, but rather the Seebeck coefficient measured by dividing the difference in voltage at room temperature and required temperature by the difference in temperatures (S=−ΔV/ΔT) that is being improved in order to obtain better efficiency [40].

In recent years, several works [41,42,43,44,45,46,47,48,49,50] which analyze and compare the performance between a thermoelectric generator and models of irreversible thermal engines have been published, showing that the best thermodynamic working conditions are obtained when the relative current density is equal to a specific value which directly depends on the material properties [18]. The traditional mechanism to increase the efficiency of TEG devices focuses on improving the thermoelectric properties of materials; meanwhile, little has been studied about the influence of TEG device configuration and optimization on its performance. In this line, Chen et al. accomplished enhancing the power output by about 34.6% [51]. In this work, an analysis of TEG devices considering two objective functions that were proposed within the field of finite time thermodynamics, namely, the efficient power and the Omega function, is presented. Two particular cases will be analyzed in more detail: the exoreversible model and the so-called endoreversible case. Although the results obtained are based on models with a greater or lesser degree of idealization, the usefulness lies in the fact that it provides qualitative information on the optimal performance of these devices under the addressed criteria.

This paper is organized as follows. Firstly, in Section 2, the configuration of a thermoelectric generator (TEG) and the optimal criteria are presented. Secondly, Section 3 briefly displays the case when only internal irreversibilities are considered. The exoreversible case is studied in Section 4; meanwhile, in Section 5, the endoreversible case is analyzed. Finally, concluding remarks are enunciated in Section 6.

## 2. Thermoelectric Model and Optimal Criteria

### 2.1. Thermoelectric Generator

**Figure 1:**
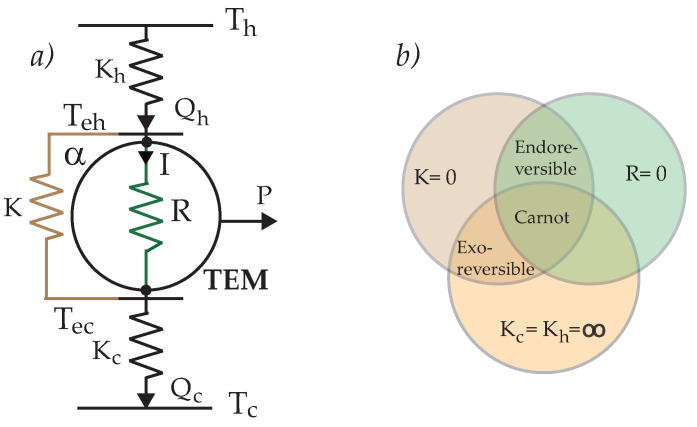
(**a**) Scheme of the TEG with internal and external irreversibilities. (**b**) Diagram of thermodynamic configurations.

The thermoelectric generator is a useful device for converting thermal energy directly into electrical energy via the Seebeck effect, and the properties of TEG are governed by the coupling of Ohm and Fourier laws. In this sense, it has the attributes of a thermal engine operating between two thermal sources. A TEG consists of two legs of thermoelectric materials (TEM), which are connected electrically in series and thermally in parallel. Figure 1a shows a scheme of TEG which operates between two thermal reservoirs with temperatures $T_{h}$ and $T_{c}$ ($T_{h}$≥$T_{c}$), having external thermal conductances $K_{h}$ and $K_{c}$, *R* is the internal resistance of TEM with electric current *I* flowing through α, the Seebeck coefficient, and *K*, the thermal conductance of two arms of the couple in parallel, associated with the heat leak (open-circuit conductance). Figure 1b shows a diagram with the equivalent thermodynamic configurations for the thermoelectric generator [42]. Thermoelectric performance is characterized by its figure of merit, $z=\alpha^{2}/RK$, where a fundamental fact to improve the energy conversion efficiency is to increase the Seebeck coefficient (α) while reducing internal resistance (*R*) and contributions to thermal conductivity (*K*) [43].

The coupling between the gradients of temperature and electric potential shows the emergence of various thermoelectric effects [44,45]; thereby, the net rates of heat input and heat rejection at the TEM are given by

(1) $$Q_{h}=IT_{eh}\alpha +K\left(T_{eh}-T_{ec}\right)-\frac{RI^{2}}{2}$$

(2) $$Q_{c}=IT_{ec}\alpha +K\left(T_{eh}-T_{ec}\right)+\frac{RI^{2}}{2}$$

where the first term corresponds to convective heat flow, with $T_{eh}$ ($T_{ec}$) the effective temperature of TEM at hot (cold) side (see Figure 1a). The second represents heat leak between the cold and hot sides, and the last term is the Joule heat received by each reservoir. For coupling with thermal sources, a Newtonian heat flow between a reservoir and TEM is assumed so that

(3) $$Q_{h}=K_{h}\left(T_{h}-T_{eh}\right)$$

(4) $$Q_{c}=K_{c}\left(T_{ec}-T_{c}\right)$$

The power output of the device is given by

(5) $$P=Q_{h}-Q_{c}=I\alpha \left(T_{eh}-T_{ec}\right)-RI^{2}$$

and the efficiency $\eta =P/Q_{h}$

Following, a review of the criteria under which the TEG model will be optimized, proposed in the context of the FTT is presented.

### 2.2. Optimization Criteria

For this work, two optimization criteria for the TEG model were chosen, efficient power (EP) [23] and Omega function (Ω) [21], which are briefly described.

**Efficient Power**. In 1980, Stucki [26] using a first-order irreversible thermodynamics formalism to study biochemical energetic processes, proposed an objective function defined by the product of the power output and the efficiency. Thereafter, in 2006, Yilmaz et al. proposed a new analysis criterion for the performance of thermal engines, called efficient power (EP) [23], which considers the effects on the design of heat engines, as the product of power by the cycle efficiency. This criterion was applied straightforwardly to the Carnot, Brayton, and Diesel engines and other systems such that the approach called maximum efficient power in the context of thermal engines is depicted as the best compromise between power output and efficiency, according to the following equation:

$$E_{P}=P\eta$$

Yilmaz and co-authors concluded that efficiency at maximum efficient power is always greater than efficiency at maximum power, which shows that the design parameters under these conditions lead to more efficient engines.

**Omega Function**. In 2001, A. Calvo Hernández, et al. proposed a criterion representing the best compromise between the maximum useful energy [21] and the minimum loss of useful energy for a specific energy converter. This is independent of any environmental parameter and does not require the explicit derivation of the entropy generation, such as the case where the exergy or the ecological criteria are used as objective functions [15,20]. This feature was proposed based on a unified optimization criterion which represents a trade-off between the benefits and losses of energy to any device.

The expression for Omega function [21] for a heat engine is given by

$$\Omega =\left(2\eta -\eta_{max}\right)\frac{P}{\eta }$$

where η = $\frac{P}{|Q_{h}|}$ is the efficiency of thermal engine and $\eta_{max}$ = $\eta_{C}$ is the Carnot efficiency for the case that concerns us in this paper.

## 3. Internal Irreversibilities

When it is considered that there is a reversible heat transfer between the TEM and the thermal sources and only internal irreversibilities are presented, there is a simple expression for its efficiency at maximum output power. This version of TEG operates between $T_{h}$ and $T_{c}$ temperatures (see Figure 1a), where the input and rejection heat flows are given by

(6) $$Q_{h}=IT_{h}\alpha +K\left(T_{h}-T_{c}\right)-\frac{RI^{2}}{2}$$

and

(7) $$Q_{c}=IT_{c}\alpha +K\left(T_{h}-T_{c}\right)+\frac{RI^{2}}{2}$$

and thus the power output is

(8) $$P=I\alpha T_{h}\eta_{C}-RI^{2}=I^{2}R_{L}$$

where $\eta_{C}=1-\frac{T_{c}}{T_{h}}$ is the Carnot efficiency and $R_{L}$ the load resistance [44,52]. While the efficiency reads

(9) $$\eta =\frac{I\alpha T_{h}\eta_{C}-RI^{2}}{IT_{h}\alpha +KT_{h}\eta_{c}-\frac{RI^{2}}{2}}.$$

Solving dP/dI=0 for *I*, and substituting its value into the efficiency, Equation (9), yields the maximum power efficiency, $\eta_{P}^{I}$, that reads

(10) $$\eta_{P}^{I}=\eta_{C}\frac{\frac{m}{2}}{1-\frac{\eta_{C}}{4}+\frac{2}{zT_{h}}}$$

where $z=\frac{\alpha^{2}}{RK}$ is the figure of merit for the device and, $m=\frac{R_{L}}{R}$, as was previously reported [43].

It is also possible to find an expression for the efficiency at maximum Omega function, $\eta_{\Omega }^{I}$, given by

(11) $$\eta_{\Omega }^{I}=\eta_{C}\frac{m}{4-\frac{3\eta_{C}}{2}+\frac{{\left(\eta_{C}-4\right)}^{2}}{zT_{h}}}$$

The expression for efficiency at maximum efficient power for this case is cumbersome, so it is not presented explicitly. As previously mentioned, this work focuses on analyzing the performance of the TEG in two configurations: (1) the so-called exoreversible case and (2) the endoreversible limit, under the regimens of maximum efficient power and maximum Omega function.

## 4. Exoreversible Case

In this scenario, irreversibilities originate only within the TEM, through the internal resistance *R* [42]. The operating temperatures are those of the thermal baths, $T_{h}$ and $T_{c}$, and irreversibilities due to heat leak are not considered, that is, K=0, thus the input and output heats (See Figure 1b), are, respectively,

(12) $$Q_{h}=IT_{h}\alpha -\frac{RI^{2}}{2}$$

(13) $$Q_{c}=IT_{c}\alpha +\frac{RI^{2}}{2}$$

then the power output at this case, is again given by the expression (8). The efficiency is given by

(14) $$\eta =\frac{2\alpha \eta_{C}T_{h}-2RI}{2\alpha T_{h}-RI}$$

### Optimization

The efficiency at maximum power output, $\eta_{P}^{II}$, for this case, has been studied previously by several authors, and here is shown:

(15) $$\eta_{P}^{II}=\frac{\eta_{C}}{2-\frac{\eta_{C}}{2}}.$$

This result, as the authors point out, is found in conditions of strong couplings [42,53], whereas the efficiency at maximum efficient power is given by

(16) $$\eta_{EP}^{II}=3-\sqrt{9-4\eta_{C}}$$

The efficiency at maximum Omega function for this configuration reads as

(17) $$\eta_{\Omega }^{II}=\frac{\left(\eta_{C}-3\right)\eta_{C}}{\frac{3}{2}\eta_{C}-4}$$

**Figure 2:**
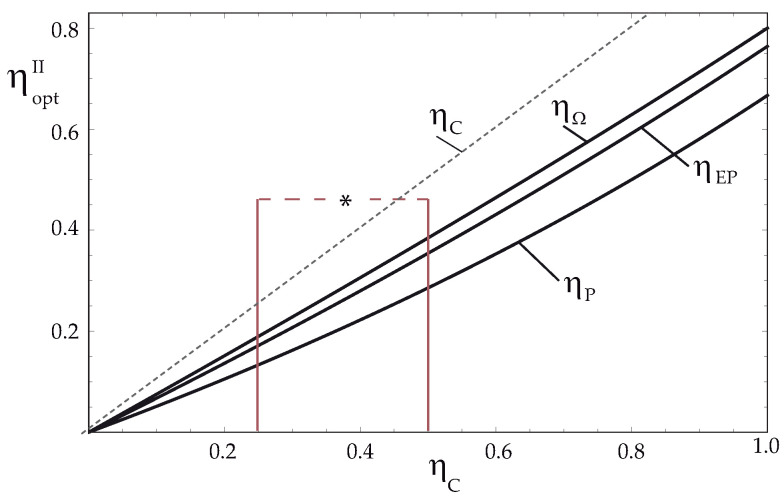
Optimum efficiencies, $\eta_{\Omega }^{II}$, $\eta_{EP}^{II}$ and $\eta_{P}^{II}$ vs. $\eta_{C}$ for exoreversible TEG configuration. * Range of operation for some TEG devices [54].

The behavior of three optimal efficiencies in this case are plotted in Figure 2. Additionally, the range of operation of $\eta_{C}$ for some TEG devices is shown and indicated with an asterisk for some applications in space systems [43,54,55]. From the results obtained for the optimal efficiencies in this configuration, it is observed that the efficiency in the maximum Omega function regime is greater than those provided at maximum efficient power and maximum output power, that is

(18) $$\eta_{\Omega }^{II}>\eta_{EP}^{II}>\eta_{P}^{II}$$

Additionally, it is important to note that the results for the optimal efficiencies are independent of the parameters of *R* and α of TEG, that is, efficiencies only depend on the temperatures of the thermal sources. This feature is common in models of conventional thermal engines under an exoreversible configuration.

On the other hand, it is known that the second law of thermodynamics imposes restrictions on the amount of energy that can be converted into useful work and indicates that, if a system undergoes an irreversible process, there is dissipation of energy, so another aspect to consider within thermal energy converters is the dissipation. Perhaps in thermoelectric generators, a study of dissipation is not substantial due to their operating conditions that produce low efficiencies. However, an analysis of dissipation in each of the regimes is given below.

The dissipation or “loss power” [20] is given by

(19) $$\phi =T_{c}\Delta S=T_{c}\left(\frac{Q_{c}}{T_{c}}-\frac{Q_{h}}{T_{h}}\right)$$

Their optimal values for each of the operating regimes are

(20) $$\Phi_{P}=\frac{\eta_{C}^{2}\left(2-\eta_{C}\right)}{4}$$

in conditions of maximum power,

(21) $$\Phi_{EP}=\frac{\left(\eta_{C}-2\right)\left(2\eta_{C}-9+3\sqrt{9-4\eta_{C}}\right)}{2}$$

for the dissipation at maximum power efficient, and

(22) $$\Phi_{\Omega }=\frac{\left(2-\eta_{C}\right)\eta_{C}^{2}}{{\left(\eta_{C}^{2}-4\right)}^{2}}$$

at maximum Omega function, where $\Phi =\phi /\frac{\alpha^{2}T_{h}^{2}}{2R}$.

**Figure 3:**
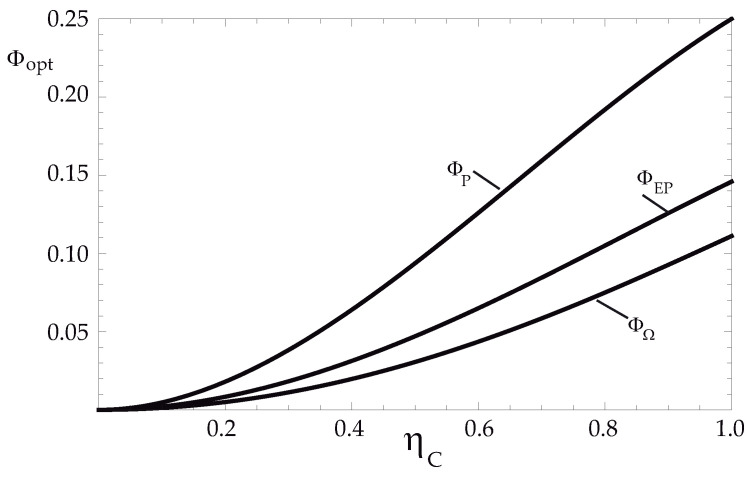
Normalized optimal dissipation Φ vs. $\eta_{c}$, under the three operating regimes.

Figure 3 shows the behavior of dissipation in each of the regimes. As can be seen, for all values of $\eta_{c}$, the dissipation satisfies that

(23) $$\Phi_{P}>\Phi_{EP}>\Phi_{\Omega }$$

From (18) and (23) it can be concluded that the operation for the TEG exoreversible configuration under the maximum Omega function provides higher efficiencies than the case at maximum power, but, on the other hand, it also generates less dissipation. Therefore, it can be considered that the Omega function is an objective function that has advantages over the other two operating regimes.

## 5. Endoreversible Limit

In this section, the optimization of the TEG in the endoreversible limit is shown, although it may lack technological importance because it is a limit where most of the sources of irreversibility vanish and only sources of irreversibility are considered in the junction between the device and the thermal baths, which are taken into account via finite-rate heat transfer [42,46]. In fact, the figure of merit, *z*, that characterizes TEGs is no longer defined in this limit. Furthermore, this case is useful because it provides explicit simple upper bounds but for non-reversible cases.

The endoreversible configuration is had when no heat leaks (K=0) and no Joule heating R=0 are considered (See Figure 1). Then

(24) $$Q_{h}=\alpha T_{eh}I=K_{h}\left(T_{h}-T_{eh}\right)$$

(25) $$Q_{c}=\alpha T_{ec}I=K_{c}\left(T_{ec}-T_{c}\right)$$

Solving for effective temperatures $T_{eh}$ and $T_{ec}$

(26) $$T_{eh}=\frac{K_{h}T_{h}}{K_{h}+\alpha I};\;T_{ec}=\frac{K_{c}T_{c}}{K_{c}-\alpha I}$$

so, the power and efficiency are expressed as

(27) $$P=\alpha K_{h}T_{h}I\left(\frac{1}{K_{h}+\alpha I}+\frac{\sigma (\eta_{c}-1)}{k_{h}\sigma -\alpha I}\right)$$

and

(28) $$\eta =\frac{\alpha I\left[1+\sigma (1-\eta_{c})\right]-\sigma K_{h}\eta_{c}}{\alpha I-\sigma K_{h}}$$

respectively, where $\sigma =\frac{K_{h}}{K_{c}}$ is the quotient of the conductances between the thermal sources and the TEM.

### Optimization

Following the same procedure as in the above section, the optimal efficiencies for endoreversible limit are found. The critical value for current *I* for our objective functions—maximum power, maximum efficient power and maximum Omega function—are obtained and substituted in the efficiency, with the following results.

Efficiency at maximum power output

(29) $$\eta_{P}^{III}=1-\sqrt{1-\eta_{C}}=\eta_{CA}$$

This result agrees with the Curzon and Ahlborn efficiency for a heat engine as expected [19], and as previously reported by [46]. The efficiency at maximum efficient power is

(30) $$\eta_{EP}^{III}=\frac{1}{4}(3+\eta_{C}-\sqrt{\left(1-\eta_{C}\right)\left(9-\eta_{C}\right)}$$

and, finally, the efficiency at maximum Omega function is given by

(31) $$\eta_{\Omega }^{III}=1-\sqrt{\frac{\left(\eta_{C}-2\right)\left(\eta_{C}-1\right)}{2}}$$

**Figure 4:**
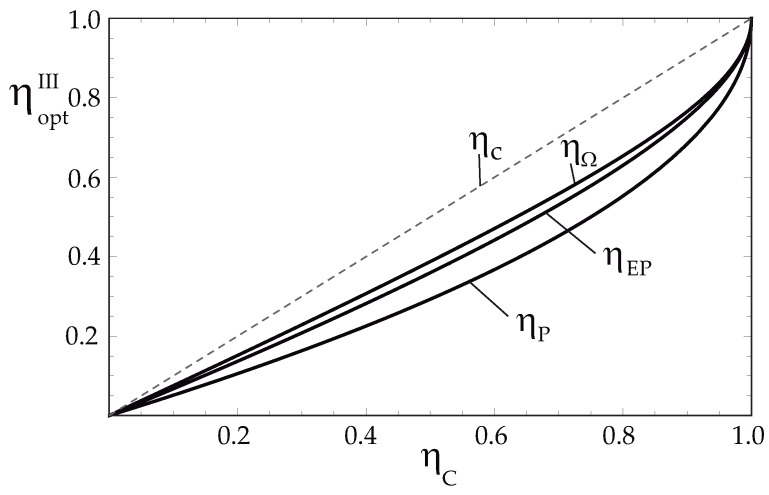
Optimal efficiencies, $\eta_{\Omega }^{III}$, $\eta_{EP}^{III}$ and $\eta_{P}^{III}$ vs. $\eta_{C}$ for endoreversible TEG limit. Carnot efficiency, $\eta_{C}$ is plot for comparison.

The behavior of these efficiencies is shown in Figure 4. As can be seen, the efficiency at maximum Omega function is always greater for all values of $\eta_{C}$ than those obtained in the regimes of maximum efficient power and maximum output power. That is

(32) $$\eta_{\Omega }^{III}>\eta_{EP}^{III}>\eta_{P}^{III}$$

as in the exoreversible configuration.

As can be seen, all the optimal values of the efficiencies are independent of σ value, that is, independent of the value of $K_{h}$ and $K_{c}$. This result is also observed in conventional thermal engines, when considering that the heat transfer between the thermal sources and working substance follow a linear heating law [56]. The dissipation for this case is not presented, but it is straightforward to show that it fulfills the same behavior as in the exoreversible case, namely, $\Phi_{P}>\Phi_{EP}>\Phi_{\Omega }$.

**Figure 5:**
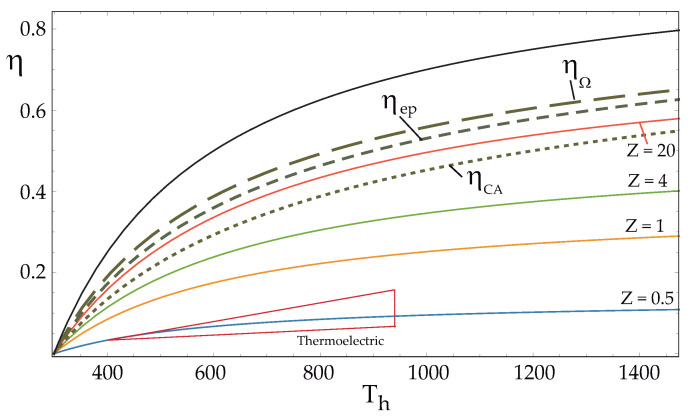
Efficiency of a TEG vs. $T_{h}$ for different values of the parameter $Z=zT_{h}$ (solid lines) given by Equation (10), with m=1.3 and $T_{C}=300$ K. The optimal efficiency for the endoreversible case is also reported for comparison (dashed lines). The red triangle indicates the area of the current efficiencies of some TEGs.

To conclude this section, Figure 5 is depicted, which shows the evolution of the efficiency of a TEG device (with all internal irreversibilities) with respect to the temperature of the hot source $T_{h}$, considering the temperature of the cold source as $T_{c}=300$ K for different values $Z=zT_{h}$ as indicated. This figure completes the one presented by Shakouri [40] (Figure 2 therein), where only the efficiency at maximum power in the endoreversible limit is given. For comparison purposes, the three optimal efficiencies found for the endoreversible limit are included. As shown, both the efficiency at maximum Omega function and maximum efficient power are greater than those that could be achieved for the value of Z=20. The Carnot efficiency, $\eta_{C}$, is also plotted. A small red triangle indicates the current range of operating temperatures and efficiencies for some TEG devices [47].

## 6. Conclusions

This paper analyzed the performance of a thermoelectric generator model with three sources of irreversibilities. The techniques arising within the field of finite time thermodynamics were applied; two objective functions were considered to optimize the performance of the TEG: the efficient power and the Omega function. In particular, two simplified models of the TEG were considered: the exoreversible case, when only internal irreversibilities from the Joule heat dissipative effect were taken into account, and the endoreversible limit, assuming that there are no internal irreversibilities and the losses are only a consequence of the couplings between the thermoelectric mechanism and the thermal sources, where a linear heat transfer law was considered.

From the results obtained, it is concluded that the efficiency under the maximum Omega function is always greater than those obtained at maximum power and maximum efficient power for both the exoreversible and endoreversible cases of TEG. Based on this, the Omega function represents an optimization criterion with advantages over the other two criteria presented in this work. In the same way, it was also demonstrated that the dissipation under this criterion is always less for the exoreversible case.

As was pointed out, improving the value of figure of merit *z* for the materials used in the assembly of thermoelectric generators is an imperative goal. Moreover, if it were possible, making them operate under any one operating regime, such as those studied here, may be a strategy that also improves the performance of these valuable devices.

## Data Availability

Not applicable.
